# Imported Malaria in the Material of the Institute of Maritime and Tropical Medicine: A Review of 82 Patients in the Years 2002–2014

**DOI:** 10.1155/2015/941647

**Published:** 2015-09-16

**Authors:** Anna Kuna, Michal Gajewski, Beata Szostakowska, Waclaw L. Nahorski, Przemyslaw Myjak, Joanna Stanczak

**Affiliations:** ^1^Department of Tropical Parasitology, Institute of Maritime and Tropical Medicine in Gdynia, Medical University of Gdansk, Poland; ^2^Department of Tropical and Parasitic Diseases, Institute of Maritime and Tropical Medicine in Gdynia, Medical University of Gdansk, Poland; ^3^Department of Infectious Diseases, Medical University of Gdansk, Poland

## Abstract

Malaria is, along with tuberculosis and HIV/AIDS, one of the three most dangerous infectious diseases in the world. In the absence of native cases since 1963, malaria has remained in Poland an exclusively imported disease, mainly occurring in people travelling to tropical and subtropical areas for professional reasons. The aim of this study was the epidemiological and clinical analysis of 82 patients admitted to the University Center for Maritime and Tropical Medicine (UCMTM), Gdynia, Poland, with a diagnosis of malaria between 2002 and 2014. The “typical” patient with malaria was male, middle-aged, returned from Africa within the preceding 4 weeks, had not used appropriate chemoprophylaxis, and had not applied nonpharmacological methods of prophylaxis, except for window insect screens. *P. falciparum* was the most frequent species. The most common symptoms included fever, shivers and intensive sweating, thrombocytopenia, elevated creatinine, LDH, D-dimers and CRP, hepatomegaly, and splenomegaly. Within the analyzed group, severe malaria according to WHO standards was diagnosed in 20.7% of patients. Our report presents analysis of the largest series of patients treated for imported malaria in Poland.

## 1. Introduction

Malaria is, along with tuberculosis and HIV/AIDS, one of the three most dangerous infectious diseases in the world. It is transmitted by the* Anopheles* mosquitoes in over 100 countries. The population at risk represents 3.2 billion (half of the world population) people, of whom 1.2 billion are at high risk. In 2013, 198 million cases of malaria were globally noted and led to 584,000 deaths of which 78% were children under five years of age [1]. In the absence of native cases since 1963, malaria has remained in Poland an exclusively imported disease [2]. Fifty cases are annually reported in Poland, while 12000–15000 cases are reported each year in the whole European Union [3]. Annually, 10–15 million people travel from Europe to the regions endemic for malaria. The popularity of exotic travels has also been continuously on the rise in Poland, but there is no precise data regarding the number of people travelling to the areas endemic for malaria. In 2013, 3.4 billion people were living in the regions where the disease occurs. The greatest morbidity and mortality have been recorded in the sub-Saharan Africa and 90% of malaria-related deaths are noted in this region [4]. In Europe, many cases of imported malaria have been recorded in people visiting friends and relatives (VFR) living in endemic areas. The Polish society is rather homogenous and the VFR group is not numerous. Malaria infections mainly occur in people traveling to tropical and subtropical areas for professional reasons (mainly missionaries as well as contract employees in Africa) and for tourism. These groups present high reluctance towards pharmacologic malaria prevention measures and, to some extent, towards nonpharmacological methods preventing mosquito bites. The abovementioned facts, combined with the delay in diagnosis and the implementation of treatment, frequently result in infected patients being admitted to hospital in advanced stages of the disease [5]. For this reason, the malaria mortality rate is several times higher in Poland compared to other European countries [2].

The aim of this study was the epidemiological and clinical analysis of patients admitted to the University Center for Maritime and Tropical Medicine (UCMTM), Gdynia, Poland, with a diagnosis of malaria between 2002 and 2014, considering the prophylaxis used, clinical symptoms at admittance and the time of their persistence prior to adequate diagnosis, and abnormalities in clinical examination and laboratory results, as well as applied treatment and its effectiveness.

## 2. Material and Methods

A retrospective audit of medical records of patients treated for malaria in UCMTM in the years 2002–2014 was undertaken. The data of 82 patients was collected from the medical records archived in the hospital (clinic) and the Department of Tropical Parasitology (results of parasitological examination). The diagnosis of malaria was based on clinical symptoms, the epidemiological (travel) history and the positive results of microscopic examination of the Giemsa-stained thick and thin blood smears (“gold standard”), the rapid diagnostic tests (RDTs), and PCR assay. All patients diagnosed with malaria were hospitalized: there was no case of outpatient treatment. RDTs (OptiMAL-IT, Bio-Rad) were performed at admittance as accessory tests for quick diagnosis, especially on nonworking days when the obtaining result of the microscopic examination was delayed. The nested-PCR with species-specific primers was used to differentiate* Plasmodium* species when microscopic examination result was inconclusive or in cases when malaria was suspected in spite of negative microscopic examination results [6, 7]. The demographic, clinical, and laboratory data was entered into a standardized data collection form and entered into an anonymous database.

*Ethical Approval*. The research was conducted in accordance with the Declaration of Helsinki. The approval number NKBBN/33/2015 of the Independent Bioethics Commission for Research of the Medical University of Gdansk has been obtained for the research.

## 3. Results

Between 2002 and 2014, 82 patients were admitted to UCMTM with malaria. Among them, 11 were female (13.4%) and 71 male (86.6%). The average age was 39.9 years, ranging between 11 and 70 years. The length of hospitalization was 3 to 44 days, 11.4 days on average. Patients returned mainly from Africa (64 cases), Asia (10 cases), and Central and Southern America (7 cases). Details are presented in Table 1.

The average time of the sojourn (in weeks) in the area endemic for malaria was 59.9 weeks, ranging between 0.5 weeks and 520 weeks (10 years).

Professional duties were declared as the purpose of travel in 37 cases and private matters in 28 cases and 17 patients did not declare the reason for travel. The details concerning purpose of travel are presented in Table 2.

The average time between the date of return from area endemic for malaria and the onset of symptoms was 16.5 days, ranging between 0 (“directly from the airport”) and 164 days.

The pharmacological prophylaxis was correctly used (appropriate drug and dosage) in 14 (17%) patients. The usage of window mosquito screens, the common equipment in the tropic, was the most popular among nonpharmacologic methods. As many as 14 patients did not use any prophylaxis against malaria. The details of prophylaxis used are presented in Tables 3(a) and 3(b).

A history of malaria in the past was reported by 25 patients. There is no data concerning the species of the previous infections. Among the 25 patients, 13 were diagnosed with* P. falciparum* and 8 with* P. vivax*, 1 was diagnosed with* P. ovale* and 1 with* P. malariae*, and 2 had a mixed infection with* P. falciparum* and* P. vivax*. In further 4 cases of* P. falciparum*, 1 case of* P. vivax*, 1 case of mixed* P. vivax* and* P. ovale* infection, and all cases without identification of species, it is unknown whether the patients had malaria in the past. In all these cases, we cannot completely exclude the possibility that the observed infection was a relapse.

The average time between the onset of symptoms and the correct diagnosis was 6.6 days (1 day to 28 days).

Prior to admittance, the majority of the patients reported fever (91.5%), shivers (42.7%), increased sweating (22%), weakness (18.3%), influenza-like symptoms (18.3%), and headaches (18.3%). The summary of all reported symptoms is presented in Table 4.

62 patients received no treatment before hospitalization. There is no data concerning 4 patients. Three patients initiated correct antimalarial treatment (appropriate drug and dosage) on their own and 11 used it incorrectly. These medications were obtained by the patients' own means. Antibiotics (at least one) were taken by 13 persons.

During the hospitalization, various clinical symptoms were observed (Table 5) and, in the cases of preceding hospitalization in other hospitals, initial diagnoses were known. Most frequent findings included fever (91.5%), hepatomegaly (63.4%), shivers (62.2%), anemia (62.2%), splenomegaly (54.9%), and increased sweating (48.8%). The oliguria or anuria was observed in 7 patients and 4 of them required hemodialysis with subsequent full recovery of renal function (3 cases) and death in one case.

Numerous various alterations were observed in laboratory test results; they are presented in Table 6.

In 79 out of 82 patients, the species of* Plasmodium* was determined*. P. falciparum* was detected in 52 people,* P. vivax* in 23 patients,* P. ovale* in 4 patients, and* P. malariae* in 4 patients. Among these patients, 4 had a mixed infection (*P. falciparum* +* P. vivax*: 2 patients;* P. vivax* +* P. ovale*: 2 patients). Three of the patients with mixed infections (2 with* P. vivax* and* P. falciparum*; 1 with* P. vivax* and* P. ovale*) visited only one location during their travel; the details regarding travel destination of one of the patients with* P. ovale* and* P. vivax* infection are unknown. The average parasitemia was 3.16% (0.01 to 37%) and it exceeded 2% in 23 people. On average, the presence of parasites in blood was observed for 2.9 days (1 day to 7 days).

The standard diagnostic method for diagnosis and differentiation of* Plasmodium* species was the microscopic examination of thick and thin blood films. RDTs were performed in 47 patients; negative results were obtained in 6 cases. Species-specific PCR assays were performed in 38 patients.

In abdominal sonography, hepatomegaly was observed in 50 patients and splenomegaly in 44 people. Among them, 34 patients had the enlargement of both organs. The chest X-ray revealed pneumonia in 11 patients, pulmonary congestion in 7 patients, atelectasis in 4 patients, hydrothorax in 5 patients, and BOOP in 1 patient. The head CT scan was not routinely performed, it was normal in 1 patient, and it revealed massive ischemic lesions and brain edema in another patient.

The patients received various treatment regimens, usually quinine (51 patients, 64.3%) and artemisinin derivates (23 people, 28%). All patients were treated after admission to the hospital. The details of therapy are summarized in Table 7.

The treatment failure was observed in one patient: death occurred on the 4th day of hospitalization. One of the patients with tertian malaria did not take primaquine in spite of receiving such recommendation. Moreover, one patient did not receive primaquine in spite of* P. vivax* infection for unknown reason. One patient received primaquine in spite of being infected with* P. malariae* and terminated the treatment after receiving the result of PCR test (the species was not initially determined in peripheral blood smear).

In cases of severe course and concurrent superinfections, additional drugs were used including mannitol (18.3%), 1 additional antibiotic (34.1%), 2 antibiotics (3.7%), 3 antibiotics (6.1%), corticosteroids (24.4%), and catecholamines (7.3%).

In 3 patients, additional parasitic diseases were diagnosed (schistosomiasis: 1 patient;* Blastocystis hominis*: 1 patient;* Giardia intestinalis*: 1 patient). Nine patients were diagnosed with pneumonia, 3 with a urinary tract infection, and 27 with a hepatic injury; 1 was diagnosed with brain infarction; 5 were diagnosed with pulmonary embolism; 1 was diagnosed with minimal consciousness, splenic rupture, and vast ischemic lesions in the brain; 3 were diagnosed with respiratory failure, 10 with acute renal failure, 59 with thrombocytopenia, 3 with purpura, and 4 with DIC; 1 was diagnosed with brain edema and 1 with ARDS.

All patients were recommended a follow-up hospitalization one month after the discharge. 85% of the patients followed this recommendation. The relapse was observed in the 3 cases of* P. vivax* infections.

## 4. Discussion

The majority of the patients hospitalized in UCMTM due to malaria were males (86,6%) returning from Africa (75.3%). The geographic region of origin of the disease is consistent with the results of other studies: one of the authors demonstrated that 1 in 5 patients with fever returning from sub-Saharan Africa had malaria [8]. The percentage is much lower (maybe 1/100) in patients with malaise returning from Asia and Southern America.

37 patients travelled for professional reasons and 26 for private ones. The majority of patients travelled for professional reasons (45%). The declared small number of VFRs (2.4%) is probably a result of the homogeneity of Polish society and the low percentage of immigrants living in Poland. The relatively low percentage of missionaries diagnosed with malaria compared to their number of hospitalization cases after sojourn in tropic is probably a result of their self-treatment with antimalarial medication brought by them from Africa.

A very small number of patients used chemoprophylaxis; only 14 of them used an appropriate drug correctly. In general, European travelers rarely use malaria prophylaxis. The data from TropNet Europe indicates that only 60.4% of the travelers used preventive measures against malaria in the years 1999-2000. The minority of patients used an appropriate drug correctly. In our study, this rate is much lower (17%). Moreover, a significant part of the travelers stay in endemic areas for many years (missionaries in particular) and recommendations for prophylaxis are different for this group [9].

The majority of* falciparum* malaria occurs within the 3 months after return, but in some patients the disease is diagnosed within up to one year, and, in the case of non-*falciparum* malaria, even several years after return [10].

In the examined group, the average time from the return from endemic area to the onset of symptoms was 16.5 days. This time was considerably shorter for* P. falciparum* (7.8 days) and for* P. vivax* it was 35.8 days. The longest time (164 days) was recorded in a soldier returning from Afghanistan infected with* P. vivax* who had been taking chemoprophylaxis (atovaquone/proguanil) correctly. The patient stayed in Afghanistan for 8 months. One month before the return, he was treated for malaria; he did not have any documentation of the treatment. Four days before admittance, episodes of high fever and symptoms of upper respiratory tract infection occurred. At admittance, the patient presented dehydration, hepatosplenomegaly, thrombocytopenia, elevated bilirubin, CRP, and D-dimer concentration. RDT, blood smear, and PCR were positive for* P. vivax*. Treatment with quinine and doxycycline was started. After one week of treatment, primaquine was introduced and the patient was discharged home in good condition. During the follow-up hospitalization one month later, no abnormalities were observed and the blood smears for malaria were negative.

Among our patients, the average time between the onset of symptoms and the correct diagnosis was 7.7 days (1 day to 28 days) for* falciparum* malaria, for* P. vivax* it was 5.75 days (1 day to 22 days), for* P. ovale* 6.5 days (5 to 8 days), and for* P. malariae* 12.5 days (5 to 21 days). In the cases of mixed infections, the average time before the diagnosis was 2.5 days for* falciparum* +* vivax* infections and 14 days for* vivax* +* ovale* infections. Only one fatal case among our center's malaria patients during 12 years of observation proves the high experience of staff working in our hospital in the field of tropical medicine, good diagnostic resources, and the vigilance of the patients themselves who frequently come directly to the hospital suspecting malaria in them.

The diagnosis of malaria in nonendemic countries remains a challenge; therefore, the doctors' awareness and ability to select patients presenting high risk of* Plasmodium* infection should diminish the risk of severe course of the disease and death [11]. On the other hand, the lack of inclusion of malaria in differential diagnosis in patients with fever returning from tropical areas and the low awareness of the necessity of fast diagnosis may lead to unacceptable delay in treatment introduction [12]. The only death in our center was a result of the delayed diagnosis and the lack of prophylaxis against malaria: the patient stayed for 5 days in Senegal, presented influenza-like symptoms after return to Poland for over 20 days, and was treated by his general practitioner for presumed bronchitis. At admittance to the hospital,* falciparum* malaria was diagnosed with parasitemia 7.4% and multiorgan failure. Despite the immediate introduction of antimalarial treatment under the conditions of intensive care unit and the initiation of hemodialysis due to anuria, the patient died on the 7th day. Ironically, the postmortem examination revealed that the* Plasmodium* species was susceptible to all available antimalarial agents, including chloroquine.

As of now, the efforts to create standards for malaria diagnosis based on clinical symptoms have failed [13]. Among English patients, high frequency of thrombocytopenia and hyperbilirubinemia was demonstrated [14]; in the Swiss series, thrombocytopenia had posttest probability of 82% and clinically enlarged spleen 85% and these two factors combined 98% [15]. In the case of our center, the “typical” patient with malaria was male (86.6%), middle-aged (average 38.9 years) who returned from Africa (75.3%) in the preceding 4 weeks, had not used appropriate chemoprophylaxis, and had not applied nonpharmacological methods of prophylaxis, except for window insect screens. At admittance, the patient presented with fever (91%), shivers (42%), and intensive sweating (22%). Laboratory results were remarkable for thrombocytopenia (83 G/L on average), elevated creatinine (1.66 mg/dL on average), LDH (759 UL/on average), D-dimers (3062 ng/mL on average), and CRP (19.37 mg/L on average). The results of imaging scans more often revealed hepatomegaly (63%) compared to splenomegaly (55%). In the analyzed group, severe malaria according to WHO standards was diagnosed in 17 patients (20.7%) after inclusion of parasitemia over 10% as one of the markers of this form of disease [16]. With the sometimes used criterion of parasitemia exceeding 2% for patients with severe malaria in nonendemic countries, this group would be expanded to 25 (30.4%). The most frequent form in the analyzed group was renal failure and hyperbilirubinemia. The WHO criteria for diagnosis of severe malaria were used for all* Plasmodium* species.

In the available literature, dominating forms of severe malaria include cerebral malaria, renal failure, and acute lung injury (ARDS) [16].

DIC and shock are less frequent and rarely exist as an isolated disorder. Icterus (due to hemolysis) and parasitemia over 2% are the prognostic factors of development of severe malaria in spite of initially good condition of the patient [17].

In the analyzed group, we did not observe significant increase in the number of* falciparum* cases and a decrease in number of tertian malaria cases was observed, contradicting studies from many other centers in nonendemic countries [18]. In each of the years, at least 10% of cases were tertian malaria, sometimes in its severe form. Reports on severe course of* vivax* cases and possible severe course of* ovale* and* malariae* infections suggest that such course may be more frequent than previously thought and that describing non-*falciparum* malaria as “mild” may be confusing [19]. In the analyzed group, we classified only one case of* vivax* infection as severe which constitutes 4% of all cases of infection with this species.

As far as the specific groups of children and pregnant women are concerned, only 2 persons in the analyzed group were minors (11 and 17 years) and only one patient was pregnant; therefore, no conclusions may be drawn from analysis of such small groups.

Various combinations of drugs were used in our hospital. No official antimalarial drug policy currently exists in Poland. National guidelines are currently under preparation; therefore, the choice of medication was mainly based on the WHO guidelines combined with the individual experience and preference of the treating physician and the head of the department. Since 2012, import of artesunate in the parenteral form has become possible which, combined with current recommendations and difficulties in quinine acquisition in Europe, motivated us to change methods of treatment in our malaria patients. Average PCT for treatment with artemisinin derivates was 2.17 days (1 day to 5 days) compared to 3.93 (1 day to 7 days) days for quinine. Along with obvious advantages of artemisinin treatment proven in multiple clinical trials, a very important factor is the convenience of drug application for the patient and staff. Among 23 patients who received ACTs, hemolytic anemia with a decrease in hemoglobin level to 8.3 g/dL was observed in only one patient in the 3rd week after treatment and it later resolved spontaneously.

The climate conditions (the number of warm days per year) are currently not favorable for the reintroduction of malaria in Poland in spite of the presence of its vector, the* Anopheles* mosquito [2]. Although there are alarming reports from Greece and Spain where cases of domestic malaria cases have recently been observed, the national epidemiological surveillance has prevented reintroduction of the disease to Poland for the last decades.

## 5. Conclusions

Our report presents analysis of the largest series of patients treated for imported malaria in Poland. Although interest in exotic travels increases in our country, usage of appropriate prophylaxis against malaria remains low. The knowledge of malaria symptoms by physicians and obtaining thorough medical and travel history as well as taking malaria into consideration in the differential diagnosis remain crucial for correct diagnosis and may help avoid unnecessary and potentially fatal delay in the introduction of appropriate treatment.

## Figures and Tables

**Table 1:** The travel destinations of patients suffering from malaria.

Continent	*n*/%	Number of persons from various countries
Africa	64/75.3	Nigeria, 11; Ghana, 4; Cameroon, 4; Sudan, 4; Kenya, 3; Angola, 2; Mali, 2; Sierra Leone, 2; Ivory Coast, 2; Congo, 2; RCA, 2; Uganda, 2; Burkina Faso, 1; Chad, 1; Ethiopia, 1; Guinea, 1; Madagascar, 1; Malawi, 1; Mozambique, 1; Senegal, 1; Tanzania, 1; Africa: travel through multiple countries, 15

Central and Southern America	7/8.2	Venezuela, 2; Brazil, 1; French Guiana, 2; Peru, 1; travel through multiple countries, 1

Asia	10/12.2	India, 6; Indonesia, 2; Afghanistan, 1; multiple countries, 1

No data	1/1.2	

*n*: number of persons.

**Table 2:** Purpose of travel of the patients.

Purpose of travel	Details of sojourn	*n*	%
Duty	Missionaries	9	10.9
	Sailors, oil platform employees	9	10.9
	Contract workers (speleologists, drillers, and heavy equipment operators)	6	7.3
		1	1.2
	Students	1	1.2
	Volunteers	3	3.6
	Soldiers	8	9.6
	Total	**37**	**45.1**

Private	VFR	2	2.4
	Tourism	26	31.7
	Total	**28**	**31.5**

Unknown		17	20.8

*n*: number of persons.

**(a):** 

Type of prophylaxis	Details		*n*	%
Chemoprophylaxis	Correctly used	Mefloquine, later followed by atovaquone + proguanil	2	2.4
		Atovaquone + proguanil	9	10.9
		Atovaquone + proguanil, later followed by doxycycline	1	1.2
		Mefloquine	2	2.4
	No chemoprophylaxis		47	57.3
	Medication taken irregularly		6	7.3
	Inappropriate medication		2	2.4
	No data		16	19.5

Window insect screens	Declared usage		39	49.6
	Not used		27	35.4

Mosquito bed nets	Declared usage		3	3.7
	Not used		63	76.8

Repellents	Declared usage		20	24.4
	Not used		46	56.1

Insecticides	Declared usage		5	6.1
	Not used		61	74.4


*n*: number of persons.

**(b):** 

	No nonpharmacologic prophylaxis	Window insect screen	Mosquito bed net	Insect repellents	Insecticides	*n*	%
Regular chemoprophylaxis	X					3	3.7
		X				1	1.2
		X			X	2	2.4
		X	X	X		2	2.4
						
						

Medication taken irregularly	X					3	3.7
		X				1	1.2
		X		X		5	6.1

Inappropriate medication	X					1	1.2
		X		X		1	1.2

No medication		X				14	17.1
		X	X			1	1.2
		X			X	3	3.7
		X		X	X	2	2.4
	X					14	17.1

*n*: number of persons.

**Table 4:** Symptoms prior to hospitalization.

Symptoms and diagnoses prior to admittance, at least one from the group present	*n*	%
Fever	75	91.5
Shivers	35	42.7
Increased sweating	18	22
Weakness, influenza-like symptoms (musculoskeletal pains, malaise), and headaches	15	18.3
Diarrhea	9	11
Vomits	7	8.5
Nausea	5	6.1
Psychomotoric retardation, confusion	4	4.9
Dyspnea, cough, oliguria or anuria, and abdominal pains	3	3.6
Weight loss, shock, respiratory infection, and loss of appetite	2	2.4
Icterus, hepatic injury, DIC, miscarriage, thrombocytopenia, anemia, and middle ear infection	1	1.2

*n*: number of persons.

**Table 5:** Symptoms and diagnoses during hospitalization.

Symptoms and diagnoses during hospitalization	*n*	%
Hepatomegaly	52	63.4
Shivers	51	62.2
Anemia	51	62.2
Splenomegaly	45	54.9
Increased sweating	40	48.8
Fever over 39°C	39	47.6
Fever 38-39°C	36	43.9
Icterus	31	37.8
Headaches	29	35.4
Renal failure	21	25.6
Muscle pains	17	20.7
Consciousness disorders	17	20.7
Nausea	11	13.4
Diarrhea	11	14.4
Abdominal pains	9	11
Shock	9	11
Vomits	8	9.8
Hearing loss	6	7.3
Bleeding	4	4.9
Respiratory failure	4	4.9
Pulmonary edema	1	1.2

**Table 6:** Abnormalities in laboratory results.

Laboratory parameter	Unit	Norm	Average value	Range of values	Mean
Hemoglobin at admittance	g/dL	11.5–16.5	13.48	6.3–16.6	12
Hemoglobin, lowest concentration			11.87	6.3–17.2	13.6
Thrombocytes	G/L	150–400	83	7–225	62
Creatinine (highest level)	mg/dL	0.5–0.9	1.66	0.57–19	1.1
GPT at admittance	U/L	<33	50.76	9–191	44
GPT highest value			89.16	9–596	54
GOT at admittance	U/L	<32	52.19	11–251	38
GOT highest value			81.51	11–842	43
LDH	U/L	<250	759.43	260–2675	559
Glucose	mg/dL	70–99	98.75	67–167	97
INR		0.8–1.2	1.26	0.9–2.89	1.2
D-dimer	ng/mL	<500	5832.79	204–20000	3062.5
pH		7.35–7.45	7.41	7.14–7.54	7.43
Sodium	mmol/L	136–145	136.15	113–146	136
Potassium	mmol/L	3.5–5.1	3.83	2.6–4.9	3.9
Bilirubin	mg/dL	<1.2	1.95	0.4–11.8	1.2
CRP	mg/L	<5	109.37	1–492	93.5

**Table 7:** The drugs used in the treatment of malaria.

Medication	Route of administration	*n*	%
Quinine in total	i.v./p.o.	51	62.2

Quinine	i.v.	47	57.4
	i.v. only	21	25.6
	p.o. only	30	36.6
	Initially i.v., followed by p.o.	26	31.7

Quinine + doxycycline		46	56.1

Quinine + clindamycin		4	4.9

Quinine + doxycycline + clindamycin		2	2.4

Artemisinin derivates in total	i.v./i.m./p.o.	23	28

Artesunate	i.v.	15	18.3
	p.o.	2	2.4

Artemether + lumefantrine	p.o.	7	8.5

b-artemether	i.m.	4	4.9

Mefloquine	p.o.	1	1.2

Sulfadoxine + pyrimethamine	p.o.	1	1.2

Primaquine	p.o.	24	29.3

Atovaquone/proguanil as adjuvant to ACTs	p.o.	5	6.1

Atovaquone/proguanil + doxycycline + primaquine	p.o.	1	1.2

Atovaquone/proguanil + quinine + doxycycline	p.o.	1	1.2

Atovaquone/proguanil + artemether/lumefantrine	p.o.	1	1.2

*n*: number of persons.

## References

[1] http://www.worldmalariaday.org/about/key-facts.

[2] Knap J., Myjak P. (2009). *Malaria w Polsce i na Świecie, Wczoraj i Dziś*.

[3] Romi R., Boccolini D., D'Amato S. (2010). Incidence of malaria and risk factors in Italian travelers to malaria endemic countries. *Travel Medicine and Infectious Disease*.

[4] World Health Organization (2011). *World Malaria Report 2011*.

[5] Santos L. C., Abreu C. F., Xerinda S. M., Tavares M., Lucas R., Sarmento A. C. (2012). Severe imported malaria in an intensive care unit: a review of 59 cases. *Malaria Journal*.

[6] Singh B., Bobogare A., Cox-Singh J., Snounou G., Abdullah M. S., Rahman H. A. (1999). A genus- and species-specific nested polymerase chain reaction malaria detection assay for epidemiologic studies. *American Journal of Tropical Medicine and Hygiene*.

[7] Myjak P., Nahorski W., Pieniazek N., Pietkiewicz H. (2002). Usefulness of PCR for diagnosis of imported malaria in Poland. *European Journal of Clinical Microbiology and Infectious Diseases*.

[8] Nic Fhogartaigh C., Hughes H., Armstrong M. (2008). Falciparum malaria as a cause of fever in adult travellers returning to the United Kingdom: observational study of risk by geographical area. *QJM: An International Journal of Medicine*.

[9] Jelinek T., Schulte C., Behrens R. (2002). Imported Falciparum malaria in Europe: sentinel surveillance data from the European network on surveillance of imported infectious diseases. *Clinical Infectious Diseases*.

[10] Bottieau E., Clerinx J., Van Den Enden E. (2006). Imported non-Plasmodium falciparum malaria: a five-year prospective study in a European referral center. *The American Journal of Tropical Medicine and Hygiene*.

[11] Jensenius M., Rønning E. J., Blystad H. (1999). Low frequency of complications in imported falciparum malaria: a review of 222 cases in south-eastern Norway. *Scandinavian Journal of Infectious Diseases*.

[12] Winters R. A., Murray H. W. (1992). Malaria—the mime revisited: Fifteen more years of experience at a New York City teaching hospital. *American Journal of Medicine*.

[13] Chandramohan D., Jaffar S., Greenwood B. M. (2002). Use of clinical algorithms for diagnosing malaria. *Tropical Medicine & International Health*.

[14] Doherty J. F., Grant A. D., Bryceson A. D. M. (1995). Fever as the presenting complaint of travellers returning from the tropics. *Quarterly Journal of Medicine*.

[15] D'Acremont V., Landry P., Mueller I., Pecoud A., Genton B. (2002). Clinical and laboratory predictors of imported malaria in an outpatients setting: an aid to medical decision making in returning travellers with fever. *The American Journal of Tropical Medicine and Hygiene*.

[16] World Health Organization (2014). Severe malaria. *Tropical Medicine & International Health*.

[17] Whitty C. J. M., Chiodini P. L., Lalloo D. G. (2013). Investigation and treatment of imported malaria in non-endemic countries. *British Medical Journal*.

[18] Health Protection Agency (2012). Malaria imported into the United Kingdom in 2011: implications for those advising travelers. *Health Protection Report*.

[19] Kochar D. K., Das A., Kochar S. K. (2009). Severe *Plasmodium vivax* malaria: a report on serial cases from Bikaner in northwestern India. *The American Journal of Tropical Medicine and Hygiene*.

